# Protection of cortical cells by equine estrogens against glutamate-induced excitotoxicity is mediated through a calcium independent mechanism

**DOI:** 10.1186/1471-2202-6-34

**Published:** 2005-05-10

**Authors:** Joel Perrella, Bhagu R Bhavnani

**Affiliations:** 1 Department of Obstetrics and Gynecology, University of Toronto, Toronto, Canada; 2 Institute of Medical Sciences, University of Toronto, Toronto, Canada; 3 Department of Obstetrics and Gynecology, St. Michael's Hospital, Toronto, Canada

## Abstract

**Background:**

High concentrations of glutamate can accumulate in the brain and may be involved in the pathogenesis of neurodegenerative disorders such as Alzheimer's disease. This form of neurotoxicity involves changes in the regulation of cellular calcium (Ca^2+^) and generation of free radicals such as peroxynitrite (ONOO^-^). Estrogen may protect against glutamate-induced cell death by reducing the excitotoxic Ca^2+ ^influx associated with glutamate excitotoxicity. In this study, the inhibition of N-methyl-D-aspartate (NMDA) receptor and nitric oxide synthase (NOS) along with the effect of 17β-estradiol (17β-E_2_) and a more potent antioxidant Δ^8^, 17β-estradiol (Δ^8^, 17β-E_2_) on cell viability and intracellular Ca^2+ ^([Ca^2+^]_i_), following treatment of rat cortical cells with glutamate, was investigated.

**Results:**

Primary rat cortical cells were cultured for 7–12 days in Neurobasal medium containing B27 supplements. Addition of glutamate (200 μM) decreased cell viability to 51.3 ± 0.7% compared to control. Treatment with the noncompetitive NMDAR antagonist, MK-801, and the NOS inhibitor, L-NAME, completely prevented cell death. Pretreatment (24 hrs) with 17β-E_2 _and Δ^8^, 17β-E_2 _(0.01 to 10 μM) significantly reduced cell death. 17β-E_2 _was more potent than Δ^8^, 17β-E_2_. Glutamate caused a rapid 2.5 fold increase in [Ca^2+^]_i_. Treatment with 0.001 to 10 μM MK-801 reduced the initial Ca^2+ ^influx by 14–41% and increased cell viability significantly. Pretreatment with 17β-E_2 _and Δ^8^, 17β-E_2 _had no effect on Ca^2+ ^influx but protected the cortical cells against glutamate-induced cell death.

**Conclusion:**

Glutamate-induced cell death in cortical cultures can occur through NMDAR and NOS-linked mechanisms by increasing nitric oxide and ONOO^-^. Equine estrogens: 17β-E_2 _and Δ^8^, 17β-E_2_, significantly protected cortical cells against glutamate-induced excitotoxicity by a mechanism that appears to be independent of Ca^2+ ^influx. To our knowledge, this is a first such observation. Whether the decrease in NOS related products such as ONOO^-^, is a mechanism by which estrogens protect against glutamate toxicity, remains to be investigated. Estrogen replacement therapy in healthy and young postmenopausal women may protect against neurodegenerative diseases by these mechanisms.

## Background

A number of neurodegenerative diseases, including Alzheimer's disease (AD) have been proposed to involve a dysregulation in the brain's glutamatergic system [1-4]. In addition, high (mM) glutamate concentrations have been documented to cause neuronal degeneration in various *in vivo *and *in vitro *models [1]. Although glutamate-induced cell death is associated with both apoptotic and necrotic changes [5] the mechanism of cell death remains to be established.

Two distinct pathways for glutamate-induced cell death have been described: the excitotoxic pathway and the oxidative pathway. The excitotoxic pathway involves the overactivation of glutamate receptors that leads to both rapid and slowly triggered cytotoxic events. The rapid effects involve the activation of the N-Methyl-D-Aspartate receptor (NMDAR) that lead to a large Ca^2+ ^influx that may be detrimental to cell viability [6]. The oxidative pathway involves the breakdown of the glutamate-cystine antiporter and a drop in glutathione levels that allows for aberrant formation of reactive oxygen species (ROS) that are neurotoxic [7,8].

Although intracellular Ca^2+ ^([Ca^2+^]_i_) is necessary for a number of physiological processes, excessive amounts may lead to neuronal dysfunction and cell death. Neuronal increases in Ca^2+ ^can activate a number of enzymes, such as phospholipases, proteases, endonucleases and nitric oxide synthase (NOS). Increase in the activity of these enzymes is associated with neuronal cell death [9,10]. Excessive glutamatergic stimulation is also associated with an increase in [Ca^2+^]_i _required for neuronal NOS (nNOS) activation and nitric oxide (NO) production within the neuron and this can result in increased cell death [9,11,12]. Therefore, the maintenance of proper Ca^2+ ^homeostasis may be effective in preventing the progression of glutamate associated neuronal degeneration.

Previous studies demonstrate that estrogens are neuroprotective against the oxidative pathway of glutamate-induced cell death in a mouse hippocampal cell line, HT22 [13,14]. This finding, along with other studies describing estrogen's neuroprotective and neurotrophic action [13,15,16] support results from observational studies that exogenous estrogen use by postmenopausal women is neuroprotective [17-20].

Although estrogens are potent antioxidants [21,22] that can prevent oxidative damage in cell culture systems, recent evidence also suggests that estrogen may inhibit glutamate-induced excitotoxicity [23]. Moreover, estrogen can lower cytotoxic Ca^2+ ^influxes induced by glutamate in hippocampal cells [24,25]. Whether this Ca^2+ ^lowering, or buffering effect occurs in other neuronal cell types, such as cortical cells, that are known to be estrogen sensitive, and whether this effect is involved in estrogen's neuroprotective effects remains to be established.

In this study, the effect of 17β-estradiol (17β-E_2_) and a novel ring-B unsaturated equine estrogen with less feminizing effects [26,27] and greater antioxidant potential, Δ^8^, 17β-estradiol (Δ^8^, 17β-E_2_) [21,22] on cell viability and [Ca^2+^]_i_, following treatment of rat cortical cells with glutamate was studied. The concentrations of glutamate used in the present study are relatively lower than those used in previous studies where cell death may have occurred mainly via the oxidative pathway. It is hypothesized that both estrogens will protect cortical cells from glutamate induced excitotoxicity by modifying the initial Ca^2+ ^influx in addition to acting as antioxidants.

## Results

### Purity of neuronal cultures

**Figure 1:**
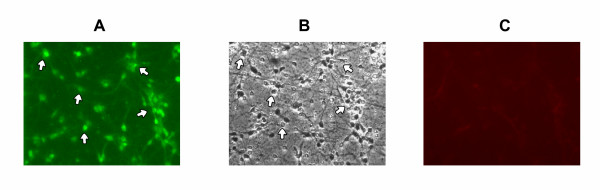
**Immunocytochemistry for neuron specific enolase (NSE) (green) and glial fibrillary acidic protein (GFAP) (red)**. Cortical cells were grown in serum-free Neurobasal medium supplemented with 2% B27 for 7 days prior to dual staining and visualization with fluorescence and phase contrast microscopy. Images shown were taken at 300× magnification. All images are taken from the same field of view. A. Fluorescence image of cortical cells stained for NSE. Arrowheads represent cells stained positive for NSE. B. Phase contrast image of cortical cells. C. Fluorescence image of cells stained for GFAP. Note almost all cells in (B) stain positive for NSE (A) and no cells stain positive for GFAP (C) indicating absence of glia.

Freshly isolated embryonic cortical cells obtained from day 17–19 pregnant rats were cultured and maintained in serum-free neurobasal medium containing B27 supplements for 7 days as described under "Materials and Methods". Phase contrast microscopy indicated that the cells had characteristic morphology of neurons and their cellular extensions (dendrites) were clearly visible (Figure 1B). Immunocytochemistry using anti-rat neuronal specific enolase (NSE), and anti-glial fibrillary acidic protein (GFAP) indicates, as can be seen in Figure 1A and Figure 1C, that the cells stained positively only for neuronal enolase (green) while GFAP staining (red) was not detectable. These data further indicate that almost all cells seen by phase-contrast microscopy (Figure 1B) were positive for neuronal enolase (Figure 1A). Thus, the cultures used in our studies are purely neuronal.

### Effect of glutamate on cell viability

**Figure 2:**
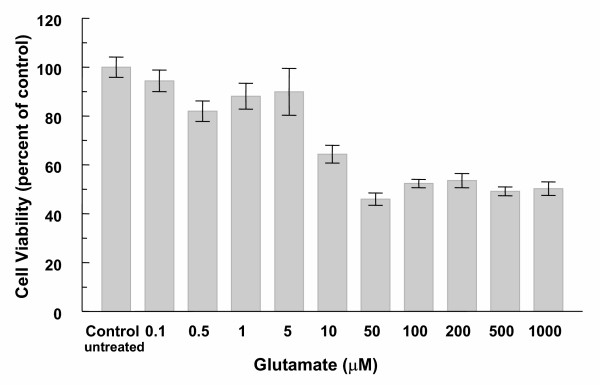
**Effect of glutamate on cell viability**. Neuronal cells were maintained for 7 days in conditions similar to those described under Figure 1. Cells were treated with various concentrations of glutamate for 20 mins. Cell viability was determined 24 hrs later. Untreated control consisted of cells exposed to Lockes buffer only. Each treatment was repeated 8 times. Error bars represent SEM.

Cortical neuronal cells (4 × 10^4^) cultured for 7 days in 96-well plates were treated with increasing concentrations (0.1 to1000 μM) of glutamate for 20 min and cell viability was measured after 24 hrs and compared with vehicle-treated samples. As depicted in Figure 2, 0.1 to 5 μM glutamate resulted in minimal cell death. As the amount of glutamate increased between 5 to 100 μM, the cell death increased gradually in a dose-dependent manner. Thus, treatment with 10 μM glutamate and 50 μM glutamate decreased cell viability by 35.6  ± 3.6% and 54 ± 2.5% respectively. Higher concentrations (>50 μM) of glutamate did not result in any further decrease in cell viability (Figure 2). Thus cell death induced by acute glutamate treatment reached a plateau in these rat primary cortical cells with concentrations of glutamate between 50 and 1000 μM. For all subsequent experiments 200 μM glutamate was used to induce cell death and this concentration resulted in a mean cell death of 48.7 ± 0.7%.

### Kinetics of glutamate-induced cell death

In order to determine the kinetics of glutamate excitotoxicity, rat primary cortical neurons were cultured for 7 days and then treated with 200 μM glutamate for 20 min. Cell death was then assessed at 2, 4, 8, and 24 hrs as described under "Materials and Methods".

**Figure 3:**
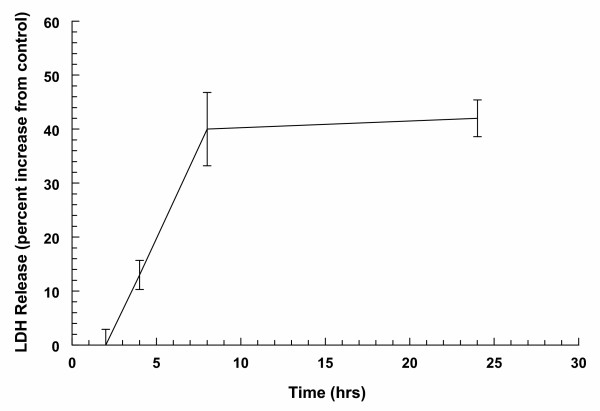
**Kinetics of glutamate-induced cell death**. Neuronal cells were maintained for 7 days in conditions similar to those described under Figure 1. Cells were treated with 200 μM glutamate for 20 mins. LDH release was measured at 2, 4, 8 and 24 hrs later. Results are expressed as percent LDH increase from control values. Each point is an average of 8 replicates. Error bars represent SEM.

The results depicted in Figure 3 indicated that acute glutamate treatment resulted in a time-dependent increase in LDH release compared to vehicle treated cells. Measurement of LDH release at 2 hours resulted in no observable difference in LDH release compared to vehicle treated samples, however after 4 hours, an increase in LDH release of 13 ± 2.7% (p < 0.05) compared to control was observed. At 8 hours LDH release increased by 40 ± 6.8% (p < 0.05) over control. No significant increase in LDH release was observed thereafter (Figure 3). The change in LDH release ratio between glutamate and the control cells at 8 hours (40 ± 6.8%) compared to 24 hrs (42 ± 3.4%) was not statistically significant. Thus, it appears that the majority of cell death in rat primary cortical cells from acute glutamate exposure occurred between 2 to 8 hours post glutamate treatment.

### Effect of estrogen on glutamate-induced cell death

**Figure 4:**
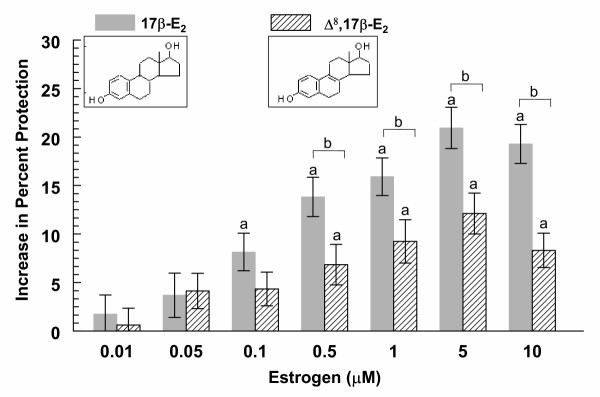
**Effect of estrogen on glutamate-induced cell death**. Neuronal cells were maintained for 6 days under conditions similar to those described under Figure 1. Cells were treated with various concentrations of 17β-E_2 _and Δ^8^, 17β-E_2_. After 24 hrs cells were treated with 200 μM glutamate for 20 mins. Cell viability was measured 24 hrs later. Results are expressed as increase in percent protection compared to glutamate alone. Solid bars represent 17β-E_2_. Hatched bars represent Δ^8^, 17β-E_2_. a. p < 0.05, between glutamate alone and estrogen treatment. b. p < 0.05, between 17β-E_2 _and Δ^8^, 17β-E_2_. Each experiment was repeated a minimum of 6 times with 8 replicates per experiment. Error bars represent SEM.

To determine whether the neurotoxic effect of glutamate can be prevented by estrogen treatment, cells were pretreated for 24 hrs with various concentrations, 0.01 to 10 μM, of 17β-E_2 _or Δ^8^, 17β-E_2_, glutamate treatment was then initiated and cell viability was measured after 24 hrs. An acute (20 minute), 200 μM glutamate exposure resulted in a 50% decrease in cell viability. As depicted in Figure 4, pretreatment with 17β-E_2 _or Δ^8^, 17β-E_2 _for 24 hrs resulted in a dose-dependent increase in cell viability against glutamate-induced neurotoxicity. No significant protection was observed with 0.01 to 0.05 μM 17β-E_2_, however, 0.1 to 5 μM 17β-E_2 _resulted in a 8.16 ± 1.93% to 20.94 ± 2.13% increase in protection (p < 0.05) respectively. Similarly, pretreatment with 0.01 to 0.5 μM Δ^8^, 17β-E_2 _resulted in an increase in protection from 0.63 ± 1.74% to 6.85 ± 2.09% respectively, however, statistical significance at these concentrations was not reached. In contrast, pretreatment with 1 and 5 μM Δ^8^, 17β-E_2 _resulted in a significant (p < 0.05) increase in protection of 9.24 ± 2.23% and 12.12 ± 2.11% respectively (Figure 4). Higher concentration (10 μM) of both estrogens did not result in any further increase in protection. Although both 17β-E_2 _and Δ^8^, 17β-E_2 _were able to significantly reduce the glutamate-induced cell death complete protection was not achieved with the concentrations tested. However, the results do indicate that 17β-E_2 _was more effective in preventing glutamate-induced cell death than Δ^8^, 17β-E_2 _(Figure 4) and this was found to be significant with the 0.1 to 10 μM concentrations of estrogen tested (p < 0.05). Pretreatment of cells with estrogen for 15 minutes did not protect the cells against glutamate neurotoxicity, (data not shown).

### Effect of NMDA and NOS inhibitors on glutamate-induced cell death

**Figure 5:**
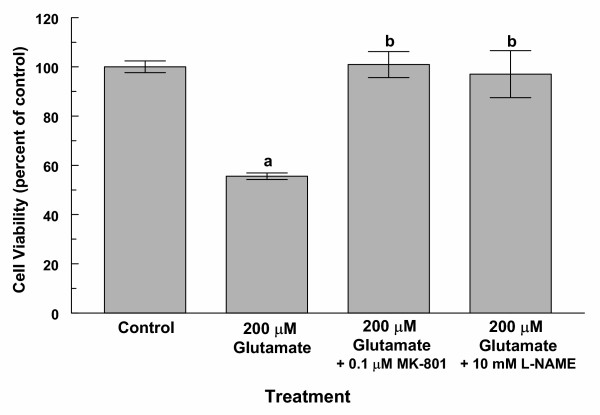
**Effect of MK-801 and L-NAME on glutamate-induced cell death**. Neuronal cells were maintained for 7 days in conditions similar to those described under Figure 1. Cells were treated with either 0.1 μM MK-801 or 10 mM L-NAME. After 15 mins cells were then treated with 200 μM glutamate. Results are expressed as percent of control. a. p < 0.05 compared to control. b. p < 0.05 compared to glutamate alone treatment. Each bar represents a mean of 8 replicates. Error bars represent SEM.

To determine whether the neurotoxic effect of acute glutamate treatment on rat primary cortical neurons is mediated through the NMDAR or NO, cell viability was determined following inhibition of NMDAR function or inhibition of NOS activity in cells treated acutely with 200 μM glutamate. As can be seen in Figure 5, a 20 minute exposure with 200 μM glutamate in rat primary cortical cells resulted in a 44.43 ± 1.34% decrease in cell viability compared to the vehicle treated control (p < 0.001). A 15 minute pretreatment with 0.1 μM MK-801, a non-specific antagonist of the NMDAR, completely prevented cell death (p < 0.001) compared to glutamate alone treated samples. Similarly, a 15 minute pretreatment with 10 mM NOS inhibitor L-NAME also completely prevented the cell death induced by glutamate (p < 0.001) compared to glutamate alone treated samples. These results clearly indicate that cell death induced by glutamate involves both NMDAR and NO.

### Effect of glutamate on intracellular calcium levels

**Figure 6:**
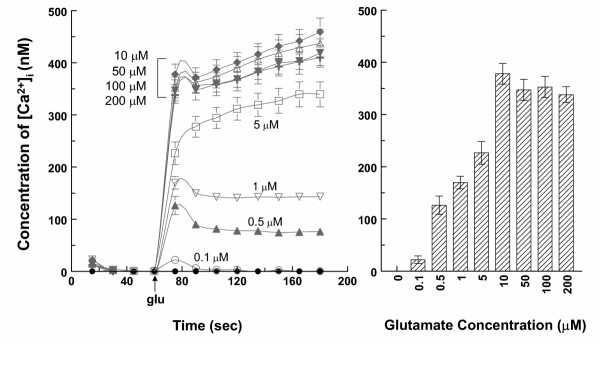
**Effect of time and concentration of glutamate on [Ca^2+^]_i_**. Neuronal cells were maintained for 7 days in conditions similar to those described under Figure 1. Cells were loaded with 5 μM Fluo-3 AM and treated with various concentrations of glutamate. Changes in fluorescence were monitored using the Fluorskan Ascent FL and results were converted to changes in [Ca^2+^]_i _(nM) as described in the materials and methods. A. [Ca^2+^]_i _measurements were made every 15 secs. Baseline levels of [Ca^2+^]_i _were monitored for 60 secs prior to injection with various concentrations of glutamate (indicated by glu). Following injection changes in [Ca^2+^]_i _were monitored for an additional 120 secs. B. Initial peak [Ca^2+^]_i _change measured at 15 secs post glutamate injection. Each point/bar represents a mean of 8 replicates. Error bars represent SEM.

To evaluate the effect of glutamate on [Ca^2+^]_i _concentrations in cortical neurons various glutamate concentrations, (0.1 to 200 μM), were added to wells and changes in relative fluorescence units (RFU) were recorded. Baseline readings were taken every 15 seconds for a total of 60 seconds prior to an injection (10 μl) of glutamate into each well (indicated by *glu *Figure 6A). Readings were continued for another 2 minutes post-injection. The kinetics of glutamate induced Ca^2+ ^influx are depicted in Figure 6A, and as can be seen, glutamate treatment (Figure 6A) results in a rapid increase in [Ca^2+^]_i _with all concentrations of glutamate (0.1 to 200 μM) tested. This initial rapid rise in [Ca^2+^]_i _occurs at 15 seconds after glutamate addition and is followed by a reduced rate of Ca^2+ ^influx. The kinetics of glutamate-induced Ca^2+ ^influx following the initial rise in [Ca^2+^]_i _remain relatively stable, but are elevated at glutamate concentration between 0.1 and 1 μM. However, at higher glutamate concentration (5 to 200 μM), [Ca^2+^]_i _continues to increase following the initial rise in [Ca^2+^]_i_. In addition, glutamate treatment results in a dose dependent increase in the initial rise in [Ca^2+^]_i _levels measured at 15 seconds, and reaches significance (p < 0.05) with 0.5 μM to 200 μM glutamate (Figure 6B). Maximal [Ca^2+^]_i _levels were observed with 10 μM glutamate and further increase in glutamate concentration did not result in any further increase in [Ca^2+^]_i _(Figure 6B).

### Effect of NMDAR antagonist, MK-801, on glutamate-induced Ca^2+ ^influx

**Figure 7:**
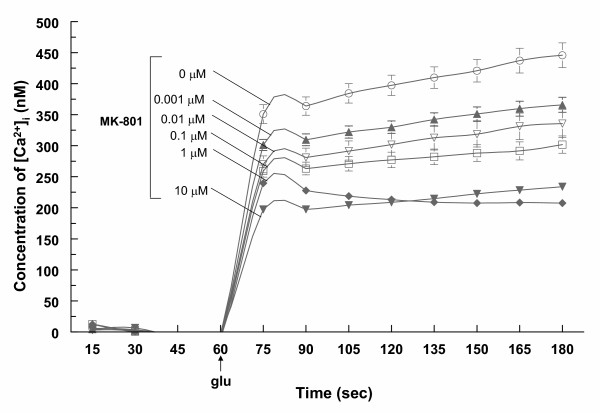
**Effect of various concentrations of MK-801 on glutamate-induced [Ca^2+^]_i _influx**. Neuronal cells were maintained for 7 days in conditions similar to those described under Figure 1, prior to loading with 5 μM Fluo-3 AM. After a 15 min treatment with various concentrations of MK-801 cells were treated with 200 μM glutamate. Changes in fluorescence were monitored using the Fluorskan Ascent FL and results were converted to changes in [Ca^2+^]_i _(nM) as described in the materials and methods. [Ca^2+^]_i _measurements were recorded every 15 secs. Baseline levels of [Ca^2+^]_i _were monitored for 60 secs prior to injection with 200 μM glutamate (indicated by glu). Following injection changes in [Ca^2+^]_i _were monitored for an additional 120 secs. MK-801 (0 μM) represents 200 μM glutamate alone treatment. Each point represents a mean of 8 replicates. Error bars represent SEM.

**Figure 8:**
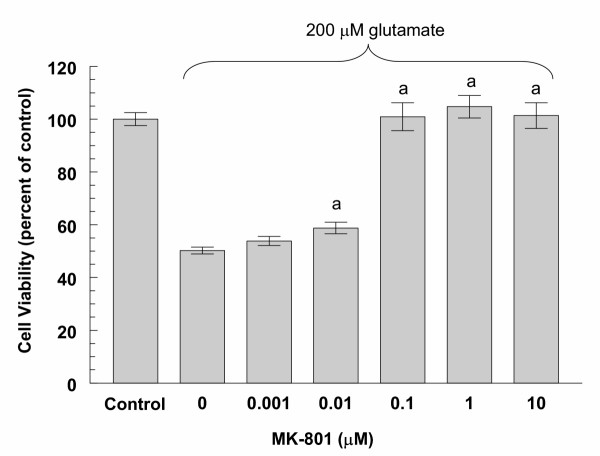
**Effect of MK-801 on glutamate-induced cell death**. Cell viability was determined in the same cells used for experiments described in Figure 7 and is expressed as percent of control. Cells were pretreated for 15 minutes with various concentrations of MK-801 (0.001 to 10 μM) and then treated for 20 minutes with 200 μM glutamate. a. p < 0.05 compared to glutamate alone. Each bar represents a mean of 8 replicates. Error bars represent SEM.

In order to evaluate the contribution of glutamate-induced Ca^2+ ^influx on cell viability the effect of NMDAR antagonist MK-801 on Ca^2+ ^influx and cell viability was measured 24 hours later in the same cell population after glutamate treatment. The maximum initial Ca^2+ ^influx was reached 15 seconds after glutamate (200 μM) injection and percent of maximum initial Ca^2+ ^influx was calculated for each concentration of MK-801 (0.001 to 10 μM). As can be seen in Figure 7, a 15 minute pretreatment with 0.001 to 10 μM MK-801 resulted in a significant dose-dependent reduction in the initial (15 seconds) Ca^2+ ^influx by 14.17 ± 2.56% to 40.30 ± 2.28% respectively. Thus, between 0 μM and 10 μM MK-801, the concentration of [Ca^2+^]_i _drops from an initial high level of 350 nM (glutamate alone) at 15 seconds to 200 nM (glutamate +10 μM MK-801). This drop in Ca^2+ ^influx was associated with a dose-dependent increase in cell viability compared to glutamate alone (Figure 8). MK-801 mediated protection reached significance (p < 0.05) with 0.01 μM MK-801 and 0.1 μM - 10 μM MK-801, completely prevented glutamate-induced cell death (Figure 8).

### Effect of estrogen on glutamate-induced calcium influx

**Figure 9:**
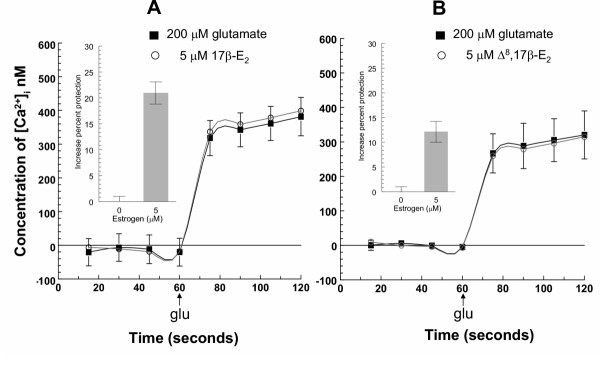
**Effect of estrogen treatment on glutamate-induced [Ca^2+^]_i _and cell death**. Neuronal cells were maintained for 6 days in conditions similar to those described under Figure 1. Cells were treated with various concentrations of 17β-E_2 _and Δ^8^, 17β-E_2_. After 24 hrs cells were loaded with 5 μM Fluo-3 AM and then treated with 200 μM glutamate. [Ca^2+^]_i _measurements were made every 15 secs. Baseline levels of [Ca^2+^]_i _were monitored for 60 secs prior to injection with 200 μM glutamate (indicated by glu). Following injection changes in [Ca^2+^]_i _were monitored for an additional 120 secs. A. Representative trace of the effect of 5 μM 17β-E_2 _on glutamate-induced [Ca^2+^]_i _influx. Inset. Increase in percent protection with 5μM 17β-E_2 _against 200 μM glutamate-induced cell death (same cells as those in which [Ca^2+^]_i _measurements were made). B. Representative trace of the effect of Δ^8^, 17β-E_2 _on glutamate-induced [Ca^2+^]_i _influx. Inset. Increase in percent protection with 5 μM Δ^8^, 17β-E_2 _against 200 μM glutamate-induced cell death (same cells as those in which [Ca^2+^]_i _measurements were made). Each experiment was repeated a minimum of 6 times with 8 replicates of each treatment condition per experiment. Error bars represent SEM.

The effect of estrogen on acute, glutamate (200 μM) induced Ca^2+ ^influx was measured at various concentrations (0.01 to 10 μM) of 17β-E_2 _and Δ^8^, 17β-E_2_. Representative traces for the effect of 5 μM 17β-E_2 _and Δ^8^, 17β-E_2 _on the glutamate-induced Ca^2+ ^influx are presented in Figure 9. Baseline [Ca^2+^]_i _measurements were made every 15 seconds for a total of 60 seconds and measurements were continued every 15 seconds following glutamate addition (indicated by *glu *in Figure 9). As shown in Figure 9, prior to glutamate addition [Ca^2+^]_i_levels were below 20 nM and remained relatively constant. Addition of 200 μM glutamate resulted in an immediate increase in concentration of [Ca^2+^]_i _to levels above 300 nM (Figure 9A and 9B). Pretreatment with 5 μM 17β-E_2 _or Δ^8^, 17β-E_2 _was not associated with any significant decrease in the levels of [Ca^2+^]_i _induced by glutamate (Figures 9A and 9B). However, pretreatment with estrogens resulted in a significant (p < 0.05) but modest increase (up to 20%) in cell protection compared to glutamate alone. Similarly, no effect on the initial glutamate induced Ca^2+ ^influx was observed at all other concentrations (0.01 to 1 μM) of estrogen. These results indicate that protection against glutamate-induced cell death in rat primary cortical neuronal cells by estrogen does not involve a reduction in the glutamate-induced, NMDAR associated, Ca^2+ ^influx, and thus estrogen mediated protection appears to be mediated by a Ca^2+ ^independent mechanism.

## Discussion

In this study, we demonstrate that in primary cultures of pure rat embryonic cortical neurons, acute (20 min) treatment with glutamate results in a dose and time-dependent cell death. Over 50% of cells die at a glutamate concentration of 50 μM. Higher concentrations (> 50 μM) of glutamate did not result in any further increase in cell death. These findings are in keeping with previous observations with cultures of purified embryonic rat spinal cord motor neurons where NMDA and non-NMDA glutamate receptor agonists kill a maximum of 40% of the neurons [28]. In contrast to these findings some studies with immortalized hippocampal cells (HT22) cultured in media containing serum have reported glutamate-induced cell death to occur only at concentrations in the millimolar range [8,13,14]. Taken together, these findings suggests that the cell type and culture conditions may play an important role in the sensitivity of cells towards glutamate-induced cell death. In addition, a main difference between rat primary cortical neuronal cells and the HT22 cells used, is that the HT22 cells are devoid of functional NMDARs and thus the cell death induced in these cells at high concentrations of glutamate is thought to proceed mainly through the oxidative pathway rather than the excitotoxic pathway [7,8].

Although the primary cortical neurons used in our study are sensitive to small changes in glutamate concentrations (0.1 to 50 μM), the extent of neuronal death under our experimental conditions (50%) is lower than that reported in a number of studies where mixed cell cultures containing neuronal, glial and other cell types were used. In these mixed cultures over 85% of motor neurons died following treatment with glutamate [29,30]. It has further been demonstrated that in such mixed cell cultures, glutamate receptor agonists may induce cell death by activating receptors on the susceptible neurons or by activating receptors in non-neuronal cells. This results in the release of substances such as NO that are toxic to neurons [31,32]. Thus, the lower extent (50%) of cell death observed in cultures maintained in neurobasal medium containing B27 supplements is most likely due to the high purity (absence of glia) of the neuronal cultures used in our study. The absence of glia in our neuronal cultures (Figure 1) maintained in serum free neurobasal medium and B27 supplements has also been reported in a number of earlier studies [16,23,33,34].

Alternatively, it is also possible that the lower extent of cell death after a short (20 min) exposure to excitotoxic levels of glutamate (200 μM) may be due to the presence of glutamate sensitive and insensitive neurons in our cultures and this has also been reported by others [23,28,35]. It has further been demonstrated in pure motor neuronal cultures (7 day old) that survival (60%) of a subset of neurons treated with glutamate was unlikely to be due to the lack of differential glutamate receptor expression [28]. These authors further suggest that selective vulnerability may be down stream of the rise in intracellular Ca^2+ ^that occurs after treatment with glutamate or agonists [28].

Previous studies, in particular with mixed cortical cultures or spinal cord neurons, have indicated that the age or maturation of neurons is important in the extent of neuronal death following treatment with glutamate. Thus, in mixed 18 day old cultures of neurons, glutamate treatment resulted in over 80% neuronal cell death compared to only 25% cell death in 4 day old cultures [36]. Since we have used cortical neurons that have been maintained in culture for only 7 days, it is possible that the extent of neuronal cell death induced by glutamate may further increase in longer-term cultures. Although we have been able to maintain some neurons for longer periods than one week, these neurons, even in the absence of glutamate, continue to die at variable rates. In keeping with our observations, Fryer et al [28] reported that 95% of pure motor neurons in culture die by 7 days. It appears that long-term cultures of pure neurons maintained in absence of serum cannot at present be used to address the question of the effect of glutamate on neurons maintained in culture for extended periods of time.

The kinetics of glutamate-induced cell death indicated that while an acute (20 minute) exposure to glutamate was sufficient to cause cell death, cell death did not occur until 4 to 8 hours following glutamate treatment. This delay in cell death is suggestive of apoptosis. Indeed, recent evidence indicates that apoptotic mechanisms can play an important role in glutamate-induced neuronal cell death [13,14,37-40]. Thus, we have demonstrated in HT22 mouse hippocampal cells that glutamate induced apoptosis was associated with DNA fragmentation, morphological changes and up-regulation of the pro-apoptotic protein Bax and down-regulation of the anti-apoptotic protein Bcl-2. Moreover, this process was reversed by estrogens [14]. We have further shown that in primary cortical neurons glutamate-induced apoptosis by caspase-3 dependent and independent mechanisms involves mitochondrial release of cytochrome C and apoptosis inducing factor (AIF) and these apoptotic changes were reversed by equine estrogens [37].

While the mechanism of glutamate-induced cell death has not been fully elucidated, results from the present study clearly indicate that glutamate-induced cell death in primary cortical cells is NMDAR and NOS linked. The relatively short time required for glutamate to initiate the cell death process and the requirement for NMDAR activation and NO production, suggest that this cell death is primarily excitotoxic. These findings are in keeping with previous observations that suggest excitotoxic cell death in cortical cultures is dependent on NMDAR and NOS activity [11,12]. Furthermore, it has been suggested that it is the production of ONOO^- ^from increased NO formation that may be responsible for cell death and that this increase in NO is dependent on NMDA associated Ca^2+ ^influx [9]. Therefore, reduction in Ca^2+ ^influx or NO production are two potential mechanisms by which neuroprotective agents may act.

The data from the present study clearly indicates that estrogen can protect against glutamate-induced excitotoxicity in primary cortical cells and a 24 hour pretreatment prior to glutamate insult is sufficient to provide this protection. In a previous study [23] protective effects of 17β-E_2 _were observed at physiological concentrations, while the concentrations we used were pharmacological. The differences are most likely due to culture conditions used: their media [23] contained antioxidants, higher cell density and lower glutamate concentrations. Since estrogens are potent antioxidants [21,22], the presence of other antioxidants in the media may explain the differences between our findings and those reported previously [23]. Although the concentrations of estrogen used in our study are high, significantly high levels [70 ng/hr/mL] were found in postmenopausal women taking a single therapeutic dose of conjugated equine estrogen [41]. Whether the levels used in the present study are attained in women taking these estrogens daily for extended periods of time remains to be determined.

It has been previously suggested that the antioxidant property of estrogen may be one possible mechanism by which estrogens exert their neuroprotective activity. Therefore, if estrogen mediated neuroprotection occurs solely through an antioxidant mechanism, then Δ^8^, 17β-E_2 _(a more potent antioxidant estrogen) [21,22,42] would be expected to be much more effective in preventing glutamate-induced cell death than 17β-E_2. _However, in the present study 17β-E_2 _was significantly more potent than Δ^8^, 17β-E_2_. The difference in neuroprotective potency suggests that the protective effect under the experimental conditions used in the present study are not solely dependent on the estrogen's antioxidant potency. The difference may also be due to the experimental design used in the present study (*ex in vivo *versus whole cell). Although Δ^8^, 17β-E_2 _is less potent than 17β-E_2_, its use as a potential neuroprotective agent warrants further investigation. This estrogen is a weaker uterotropic estrogen than 17β-E_2 _and its effects appear to be mediated mainly via the estrogen receptor β (ERβ) [26,27,43]. Thus, Δ^8^, 17β-E_2 _may have therapeutic applications in both women and men for prevention of neurodegeneration. Whether this neuroprotection also occurs through the well characterized genomic mechanism involving ERs (ERα and ERβ) or through the putative membrane receptor pathways [44] remains to be investigated.

As we have shown, glutamate-induced excitotoxicity is dependent on NMDAR activity and therefore, changes in Ca^2+ ^influx across the cell membrane may trigger mechanisms involved in cell death [6]. Indeed, it was observed in the present study that following glutamate stimulation, a rapid dose-dependent increase in [Ca^2+^]_i _occurs that is associated with a dose-dependent decrease in cell viability. However, stimulation with glutamate above 10 μM did not further increase [Ca^2+^]_i _again suggesting that glutamate insensitive and sensitive cells may have been present in these primary cortical cultures.

The observation that glutamate-induced Ca^2+ ^influx decreased significantly by MK-801 suggests that this to some extent occurs via NMDAR. Increasing concentrations of MK-801 resulted in a gradual lowering of the [Ca^2+^]_i _concentration within 15 seconds after glutamate treatment. Thus MK-801 (10 μM) reduced the [Ca^2+^]_i _from 350 nM to 200 nM in 15 seconds (Figure 7). Although the fall in [Ca^2+^]_i _levels between 0.01 to 0.1 μM MK-801 was less than 5% (270 nM to 260 nM respectively; Figure 7), the cell viability increased from 58% to 100%, (Figure 8). These types of glutamate-induced small changes in [Ca^2+^]_i _that result in large (total) and significant cell death of cortical/glial cell cultures have been previously observed [45]. These investigators explained this by suggesting the presence of a critical Ca^2+ ^"threshold". They further suggested that even a small reduction in NMDA receptor activation may be sufficient to reduce Ca^2+ ^influx below this critical threshold such that neural cell death is significantly reduced. Our results (Figures 6,7,8) indicate that perhaps such a threshold may also be present in pure neuronal cortical cells.

In the present study the effect of estrogen on excitotoxic Ca^2+ ^influx was investigated and although 17β-E_2 _and Δ^8^, 17β-E_2 _both provided significant protection (Figure 9), neither had any effect on the glutamate-induced Ca^2+^influx contrary to our working hypothesis. Thus it appears that the estrogen mediated protection of primary cortical cells from acute glutamate exposure does not involve a decrease in the glutamate-induced Ca^2+ ^influx, and to our knowledge this is a first such observation. In contrast to these findings a previous study in hippocampal cells cultured in media containing antioxidants, has shown that estrogen can reduce the glutamate-induced Ca^2+ ^influx by increasing mitochondrial sequestration of Ca^2+ ^[25]. This increased sequestration was attributed to the ability of estrogen to increase Bcl-2, an antiapoptotic protein that is important in mitochondrial Ca^2+ ^sequestration [46,47]. Whether estrogen modulates Bcl-2 expression in primary cortical cells in a manner similar to our previous study with HT22 cells [14] or whether mitochondrial sequestration of Ca^2+ ^occurs remains to be established. Furthermore, these investigators [25] cultured the hippocampal cells in medium that contained antioxidants and this may also explain some of the differences observed. Our results do however, indicate that the mechanism by means of which MK-801 and estrogens protect against glutamate-induced cell death, appears to be different. Further studies are needed to identify these differences.

There are many other effects of estrogen on the central nervous system that have been described, for example, estrogen can alter NOS expression throughout the brain and potentially upregulate antioxidant proteins through cyclic guanosine monophosphate linked mechanisms [48,49]. Such alterations may play a role in glutamate excitotoxicity by affecting subsequent events in the cell death pathway, downstream of the initial Ca^2+ ^influx. In addition, the rapid activation of mitogen-activated protein kinase (MAPK) by estrogen has been shown to be necessary for estrogenic protection although how and what the function of MAPK is in preventing cell death remains to be established [50]. Similarly, the phosphorylation of CREB (cAMP [cyclic adenosine monophospate] response element-binding protein) and upregulation of neurotrophic factors such as brain-derived neurotrophic factor [51], may play a role in neuronal protection [52]. Whether these factors are involved in prevention of glutamate-induced excitotoxicity by equine estrogens remains to be determined.

## Conclusion

Glutamate-induced cell death in cortical cultures occurs through NMDAR and NOS-linked mechanisms, presumably through elevated [Ca^2+^]_i _levels that activate NOS and increase the levels of NO and subsequently ONOO^-^free radicals. Equine estrogens, 17β-E_2 _and Δ^8^, 17β-E_2_, significantly protect against glutamate-induced excitotoxicity through a mechanism that is independent of Ca^2+^. This is to our knowledge a first such observation. Whether a decrease in NOS related products, such as ONOO^-^, may be one mechanism by which estrogen provides neuroprotection against glutamate toxicity, remain to be investigated.

## Methods

### Isolation and culture of cortical cells

Day 17–19 pregnant Sprague Dawley rats were anesthetized and sacrificed by cervical dislocation. The fetuses were delivered, decapitated and the brains were dissected out. Each cortex was isolated and individual cells were cultured essentially according to Brewer et al [33,34] with the following modifications. Briefly, the tissue was maintained in Hank's balanced salt solution (HBSS) (Invitrogen, Life Technologies, Burlington, ON, Canada) until enough tissue to give sufficient cell numbers for plating was collected. The tissue was then dispersed by mechanical dissociation through a series of constricted fire polished, siliconized Pasteur pipettes to obtain a suspension of single cells. The suspension was then centrifuged and the cells were washed to remove red blood cells and other impurities. An aliquot was stained with 4% trypan blue, live cells were counted using a hemocytometer and the suspension was then diluted accordingly with neurobasal defined medium (Invitrogen, Life Technologies, Burlington, ON, Canada) supplemented with 2% of B27 containing antioxidants (Invitrogen, Life Technologies, Burlington, ON, Canada), 0.5 mM glutamine, penicillin G (10 U/mL) and streptomycin (13 μg/mL) purchased all from Sigma, Canada. This medium was labeled as neurobasal complete (NBC).

Cells, 40,000 per well, were seeded onto poly-D-lysine coated 96 well plates (BD Biosciences, Discovery Labware, Bedford, MA, USA) and were maintained in NBC. Under these culture conditions, it has been previously demonstrated (16,23,33,34) that only neurons survive while glia are essentially absent. The purity of our cortical neuronal cultures is described separately. After day 1 of plating the media was completely replaced and on subsequent days 50% of the media was replaced. Cell cultures were maintained for 7–12 days at which time glutamate experiments were initiated. Preliminary experiments carried out during the standardization of the methods indicated that the cell viability decreased and was variable after day 7 in culture. Although some neurons were viable up to day 12, all experiments described in the manuscript were carried out in cultures maintained for only 7 days.

### Detection of neuronal specific enolase (NSE) and glia-specific glial fibrillary acid protein (GFAP) by immunocytochemistry

**Figure 10:**
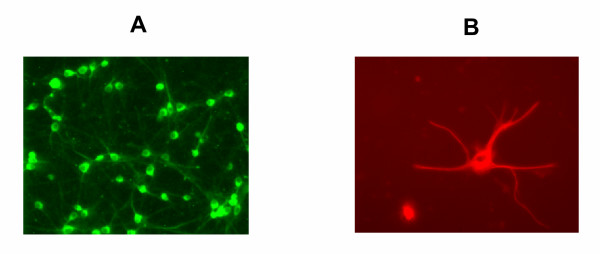
**Detection of NSE (green) and GFAP (red) by immunocytochemistry in neurons and glia**. Cells were labelled with either anti-NSE or anti-GFAP and visualized by fluorescence microscopy. Neuronal cells were grown in Neurobasal medium supplemented with 2% B27 and maintained for 7 days in culture. Glial cells were grown in DMEM supplemented with 10% Fetal Bovine Serum and maintained for 7 days in culture. (A) Neurons labelled with anti-NSE. (B) Glial cells labelled with anti-GFAP. Images were taken at 300× magnification.

Cortical cells on poly-D-lysine coated plates were cultured in NBC medium as described above. After seven days, cells were washed twice with PBS and then fixed with 4% formaldehyde in PBS for 15 minutes. Cells were then washed three times with PBS and non-specific binding was blocked for 5 mins with PBS containing 1% BSA (Sigma, Canada), 5% normal goat serum (Sigma, Canada), 0.05% Triton-X100 (BDH, Canada). Cells were incubated for 2 hrs at room temperature with rabbit anti-rat neuron specific enolase (anti NSE; Polyscience, PA, USA) 1:1000 dilution in block solution. After rinsing 4 times with PBS, the cells were incubated at room temperature with Alexa Fluor 488 F(ab')_2 _fragment of goat anti-rabbit IgG (H+L) secondary antibody (Molecular Probes, Invitrogen, Canada) 1:400 dilution in block solution. Cells were then rinsed 4 times with PBS and the fluorescence labeling was visualized and photographed using Olympus IMT-2 microscope, equipped with a mercury light source and spot slider digital camera (Diagnosis Instruments, MI, USA). The neuronal cells displayed characteristic green fluorescence (Figure 10A).

The neuronal cells maintained in NBC are devoid of glial cells and to obtain cultures containing glia, the cortical cells were prepared as described above but were maintained in Dubelcco's Modified Eagle Medium (DMEM, Sigma Canada) containing 10% Fetal Bovine Serum (FBS; Invitrogen Canada Inc; On, Canada). After 7 days in culture, the mixture of cells were processed for immunocytology by incubating with the primary antibody mouse anti-GFAP (1:4000 dilution; Sigma Canada) and then with Texas Red dye conjugated AffiniPure F (ab')_2 _fragment goat anti-mouse IgG (H+L) (1:100 dilution; Jackson Immunoresearch Laboratories, PA, USA) as described above for NSE, (data not shown). The GFAP positive glial cells stain red as seen in Figure 10B. For double staining, cells maintained in NBC medium were first incubated with primary and secondary antibodies for GFAP and after rinsing four times with PBS, the cells were incubated with antibodies for NSE. Cells were then visualized and photographed. The dual staining indicated that the cells were only positive for neuronal NSE and negative for glial GFAP (Figure 1A and 1C).

### Treatment of cells

For determination of the effect of glutamate on the viability of rat primary cortical cells, the cells were washed twice with a modified Locke's buffer (154 mM NaCl, 5.6 mM KCl, 3.6 mM NaHCO_3_, 2.3 mM CaCl_2_, 5.6 mM Glucose, 5 mM HEPES, 10 μM Glycine, pH 7.4) and incubated for 24 hours at 37°C and 5% CO_2 _in NBC medium, plus 0.5 mM glutamine and 2% B27 minus antioxidants (NBC AO-). Following this, the cells were washed 3 times with modified Locke's buffer and glutamate exposure was initiated. All glutamate treatments were conducted in modified Locke's buffer. Various concentrations of glutamate were added to a final concentration of 0.1, 0.5, 1, 5, 10, 50, 100, 200, 500 and 1000 μM for a total of 20 minutes and changes in [Ca^2+^]_i _were recorded. The cells were then washed with modified Locke's buffer and NBC AO- was added and cells were incubated at 37°C, 5% CO_2 _for 24 hours, following which cell death/viability was measured. Measurement for Ca^2+ ^and cell death/viability were performed on the same culture of cells.

The kinetics of glutamate-induced cell death following a 20 minute exposure to glutamate was also measured using a concentration of glutamate found to be toxic to 50% of the cells. Cell death measurements were made at 2, 4, 8 and 24 hours post glutamate treatment and comparisons between glutamate treated samples and vehicle (modified Locke's buffer) treated samples were made.

For the determination of the effects of estrogen on glutamate induced changes, cells were washed twice with modified Locke's buffer and then fresh NBC AO- and estrogens were added to final concentrations of 0.01, 0.05, 0.1, 0.5, 1 and 10 μM and the cells were incubated for 24 hours at 37°C and 5% CO_2_. Estrogens were dissolved in ethanol and diluted to working concentrations with NBC AO- medium. The final concentration of ethanol was 0.2% for control and estrogen treated samples. Following incubation with estrogen and prior to glutamate exposure cells were prepared for [Ca^2+^]_i _measurements as outlined below. The cells were washed and incubated in Locke's buffer containing sufficient (200 μM) glutamate (pre-determined) to give 50% cell death. Incubations were carried out at 37°C for 20 minutes and changes in [Ca^2+^]_i _were recorded. Following glutamate treatment cells were washed with fresh Locke's buffer and NBC AO- was added. The cells were again incubated for 24 hours at 37°C and 5% CO_2_, following which cell viability was determined.

In some experiments various concentrations of MK-801, an NMDAR antagonist, and 10 mM of L-NAME [53], a NOS inhibitor, were added 15 minutes prior to glutamate exposure following which changes in [Ca^2+^]_i _and cell viability were measured.

### Evaluation of cell death and viability

The Lactate dehydrogenase (LDH) cytotoxicity assay (Promega G1780, VWR, Toronto, Ontario, Canada) and the CellTiter 96^® ^AQ_ueous _Non-Radioactive cell proliferation assay (MTS method) (Promega G5430) were used to assess cell death and viability respectively. The LDH assay measures the amount of lactate dehydrogenase that is released from dead cells while the MTS method measures the formation of a formazan product in live cells. Changes in optical density (O.D.) at 490 nm were measured using the SpectraMax 340 microplate reader and SOFTmax^® ^PRO software (Molecular Devices, California, U.S.A.) and comparisons of treated to control values were made. Both of these methods provide a reliable measure of cell death/viability.

### Measurement of intracellular calcium levels

Intracellular Calcium levels were assessed using the fluorescent probe Fluo-3 AM (Molecular Probes, Eugene, OR, USA). Changes in relative fluorescence units (RFU) were monitored with the Fluoroskan Ascent FL fluorescent plate reader equipped with a micro-injection syringe pump (Labsystems, Helsinki, Finland). The protocol for intracellular dye loading was based on the method provided by the manufacturer (Molecular Probes and Labsystems Fluo-3 AM Application Note) with minor modifications. Briefly, cells were loaded with 5 μM Fluo-3 AM for 2 hours and then washed three times with Locke's buffer to remove excess dye. Cell plates were then placed in the Fluoroskan and incubated for an additional 15 minutes at 37°C to allow for complete de-esterification of dye. Measurements of relative fluorescence units (RFU) were made using an excitation and emission wavelength of 485 and 527 nm respectively. Change in [Ca^2+^]_i _(nM) was calculated by multiplying the ratio of ΔF-Fmin and Fmax-ΔF by Kd. Where ΔF is the change in RFU, Fmax is the maximum observed RFU following NP40 treatment, Fmin is the minimum observed RFU following EGTA Ca^2+ ^chelation and Kd is the dissociation constant for Fluo-3 AM, 390 nM, provided by supplier (Molecular Probes).

### Data analysis

The dose-response curves for effects of glutamate and estrogen on cell viability and [Ca^2+^]_i _were developed using Slide Write Plus V6 Software (Advanced Graphics Software, Inc., Encinitas, CA) using the non-linear curve fitting functions. Each estrogen experiment was carried out a minimum of six times with eight replicates per treatment condition and all values shown in figures are the mean ± standard error of the mean (SEM). All treatment conditions were compared to the respective controls and an effect was determined significant if p < 0.05. All statistical analyses were performed using Instat^® ^Software (Graph Pad, San Diego, CA). To establish the effect of estrogen against glutamate induced cell death and [Ca^2+^]_i_, nonparametric statistical analyses were performed using the Kruskal-Wallis nonparametric analysis of variance test with the Dunn post hoc test to compare each estrogen concentration against the glutamate alone control.

## Authors' contributions

***JP ***is a graduate student who participated in the development of the hypothesis, study design and carried out most of the experimental work and preparation of the manuscript.

***BB ***conceived the study and participated in the development of the hypothesis, the study design, and overall direction of the study and preparation of the manuscript.

Both authors have read and approved the final preparation of the manuscript and its submission to BMC Neuroscience.
